# Alternative RNA Splicing in the Pathogenesis of Liver Disease

**DOI:** 10.3389/fendo.2017.00133

**Published:** 2017-06-21

**Authors:** Nicholas J. G. Webster

**Affiliations:** ^1^Medical Research Service, VA San Diego Healthcare System, San Diego, CA, United States; ^2^Department of Medicine, School of Medicine, Moores Cancer Center, University of California San Diego, La Jolla, CA, United States

**Keywords:** non-alcoholic fatty liver disease, RNA splicing, hepatocellular carcinoma, splicing factors, microarrays

## Abstract

Non-alcoholic fatty liver disease (NAFLD) is becoming increasingly prevalent due to the worldwide obesity epidemic and currently affects one-third of adults or about one billion people worldwide. NAFLD is predicted to affect over 50% of the world’s population by the end of the next decade. It is the most common form of liver disease and is associated with increased risk for progression to a more severe form non-alcoholic steatohepatitis, as well as insulin resistance, type 2 diabetes mellitus, cirrhosis, and eventually hepatocellular carcinoma. This review article will focus on the role of alternative splicing in normal liver physiology and dysregulation in liver disease.

## Introduction

Publication of the human genome sequence in 1995, and subsequently other mammalian genomes in the following two decades, has revealed a surprisingly small number of genes that must account for tremendous species diversity. Indeed, recent estimates have suggested that the number of human protein-coding genes may be as low as 19,000 (1). This is surprising given that the *Drosophila melanogaster* and *Caenorhabditis elegans* genomes encode 17,000 and 21,733 genes, respectively (2, 3), and even the lowly amoeboflagellate *Naegleria gruberi*, a free-living unicellular eukaryotic organism, has 15,727 genes (4). These observations posed a diversity paradox for genetics and challenged the one gene-one protein hypothesis. Unlike prokaryotic and lower eukaryotic genes, most mammalian genes are composed of multiple coding exons with intervening non-coding introns of variable length. Very often these exons encode discreet protein modules or substructures. Transcription of these split genes produces a primary transcript that requires further processing to remove the intronic sequences, a process called RNA splicing. Much of our understanding of the mechanism of RNA splicing comes from elegant biochemical and genetic studies in yeast and has been extensively reviewed (5). The presence of exons and introns provides a solution to the diversity paradox by allowing assembly of different proteins by modular construction of RNA transcript isoforms through a process termed alternative splicing (6, 7). The diversity of RNA transcripts is further amplified by the use of alternative transcription start sites and polyadenylation sequences. Transcriptome sequencing has shown that upward of 90% of mammalian genes have multiple transcript isoforms, and an estimated 160,000 alternatively spliced transcripts are protein encoding (8, 9). Although 85% of these genes have a predominant RNA transcript isoform (10), the minor isoforms can have different functions and may play an important role in disease.

Alternative splicing and the generation of protein diversity have broad implications for clinical disease. It is estimated that 50–60% of 31,250 disease-causing mutations in the Human Gene Mutation Database affect splicing (11, 12). Approximately 16% of these mutations are located directly in splice sites (13), and 66% are SNPs, microdeletions, or insertions within exons. While some of these latter mutations have a pathogenic effect by altering protein sequence, a large proportion do not, but rather interfere with splicing by interrupting exonic splicing enhancers or silencers. Cancer has been termed a disease of the genome due to the accumulation of DNA damage and genetic alterations that cumulatively cause transformation and malignancy. Indeed many mutations alter protein function by creating constitutively active oncogenic proteins or disrupting tumor suppressor proteins. However, it is now increasingly recognized that many cancer-associated RNA transcripts do not result from point mutations in the RNA itself, but rather by changes in expression or function of splicing factors that regulate the ordered splicing of primary gene transcripts giving rise to aberrant expression of oncofetal isoforms with greater proliferative capacity.

## Mechanism of Alternative RNA Splicing

Much of our understanding of mechanism of RNA splicing comes from genetic studies in yeast and biochemical reconstitution experiments (5, 14). These studies have shown that the ends of an intron are aligned for excision by a complex network of RNA and protein interactions involving both splice sites in a large complex called the spliceosome. Initially the 5′ splice site is recognized by the U1 small nuclear ribonucleoprotein particle (snRNP) by base pairing of the U1 small nuclear RNA to the 5′ splice site. The U2 snRNP is then recruited to the 3′ splice site and branch point sequence by the accessory factor U2AF. The complex containing the pre-RNA, and the U1 and U2 snRNPs is called the pre-spliceosomal complex and defines the intron. This complex then recruits the U4/U5/U6 tri-snRNP, and the spliceosome undergoes a number of rearrangements including replacement of the U4:U6 duplex with a U2:U6 duplex, loss of the U4 snRNP, and displacement of the U1 snRNP on the 5′ splice site by the U6 snRNP to create the catalytically competent splicing complex. The actual splicing reaction then proceeds by two transesterification reactions first by the branch point adenine at the 5′ splice site then by the exonic terminal hydroxyl group at the 3′ splice site resulting in ligation of the two exons and liberation of an intron-lariat structure.

What defines whether an exon is recognized in a primary RNA transcript? While U1 and U2 snRNPs can interact across short introns to define the intron in typical *in vitro* splicing reactions, this interaction is much less efficient when the size of the intron increases above 250 nucleotides (15). As most introns are kilobases in length, yet the average size of an exon is ~200 nt, definition of the splice sites *in vivo* is generally thought to occur across exons rather than introns, a process termed exon definition (16). Thus, exons are defined by binding of U1 and U2 snRNP across the exon in the primary transcript followed by the long-range splice site pairing across the intron to assemble functional spliceosomes. Support for this exon-definition model comes from the finding that mutation of the downstream 5′ splice site on an exon can alter splicing of the upstream intron, so the sequential splicing of introns is coordinated and does not occur independent of each other.

How does this process allow for the use of different exons or splice sites during alternative splicing? In general, alternative exons contain weak splice sites that are not recognized efficiently (14). For genes that are co-transcriptionally spliced, this can be explained by a kinetic competition for assembly of the U1–U2 complex across alternative exons, or for non-co-transcriptionally spliced genes, this could be explained by the differences in the stability of the resulting complexes. Whether these weak exons are recognized is determined to a large extent by the presence of *cis*-acting binding sites for RNA-binding proteins within the exon or adjacent introns in the primary RNA transcript (17–19). Two of the most well-studied families of RNA-binding splicing regulators are the SR proteins (16 members) and the hnRNPs (20 members) (20–24), but there are also a number of less-studied families of RNA-binding proteins that regulate splicing, including the CELF/BRUNOL family, the Zinc-finger proteins, and the RBM family (25–27). All proteins contain RNA-binding domains allowing sequence specific-binding to RNA. Proteins of the SR family have an RNA recognition motif (RRM) at the amino-terminus, and a C-terminal domain that is enriched in arginine/serine dipeptides (RS domain) and heavily phosphorylated. hnRNP proteins show greater structural diversity than SR proteins, with RRM, RGG (arginine/glycine rich box), or KH (K homology box) RNA-binding domains. Additionally, the hnRNPs have auxiliary functional domains, which mediate protein–protein interactions and/or localization, and are divergent in protein sequence and structure (28). Both SR proteins and hnRNPs can promote or inhibit exon recognition depending on sequence context, thus modulating the usage of alternative exons or splice sites (14, 29, 30).

## Alternative Splicing in Liver Development

While liver-specific transcriptional regulation is well established and has been studied for decades, liver-specific alternative splicing is less well understood. Alternative splicing has traditionally been studied on a gene-by-gene basis, which required prior knowledge of the gene transcripts, but the development of high-throughput array and RNA sequencing (RNA-seq) technologies has allowed an unbiased assessment of alternative splicing events (Table 1) (31). In a recent study, Nellore et al. aligned 21,504 human RNA-seq samples from the Sequence Read Archive to the human genome and compared exon–exon junctions to the known gene annotation databases (32). Approximately 19% of splice junctions (56,861) that were found in at least 1,000 samples were not previously annotated, indicating that a great deal of transcript diversity is still to be discovered. Brain, liver, and testis show the greatest diversity in transcripts with ~35–40% of genes showing alternative exon or splice site usage (33).

**Table 1:** Studies reporting alterations of RNA splicing factor expression or alternative splicing in liver.

Study	Objective	Method	Reference
Ameur et al.	Nascent transcripts and co-transcriptional splicing in brain and liver	RNA sequencing (RNA-seq) on human and chimpanzee RNA from brain and liver	(31)

Nellore et al.	Alternative splicing across Sequence Read Archive	Re-aligned 21,504 RNA-seq samples from SRA	(32)

Yeo et al.	Alternative splicing across human tissues	Re-aligned cDNA and EST alignments	(33)

Bhate et al.	Alternative splicing during mouse liver development	RNA-seq on FVB/NJ mice at embryonic day 18, and postnatal days 14, 28, and 90	(39)

Peng et al.	Transcriptome and alternative splicing during liver development	RNA-seq on male C57BL/6 mice (*n* = 3) at e17, postnatal days 0, 1, 3, 5,10, 15, 20, 25, 30, 45, and 60	(40, 41)

Lake at al	Transcriptome of non-alcoholic fatty liver disease (NAFLD)	Microarrays on 10 steatotic, 9 non-alcoholic steatohepatitis (NASH) with fatty liver, 7 NASH w/o fatty liver, and 19 normal subjects	(76)

Moylan et al.	Transcriptome in NAFLD	Microarrays on 40 mild NAFLD and 32 severe NAFLD subjects	(77)

Pihlajamaki et al.	Comparison of liver transcriptomes in obese and lean humans and mice	Microarrays on 5 lean non-diabetics and 8 obese subjects undergoing bariatric surgery	(79)

Zhu et al.	Liver transcriptome and alcohol-metabolizing genes in NAFLD	Microarrays on 40 mild NAFLD, 32 severe NAFLD, 15 alcoholic hepatitis, and 7 normal subjects	(83)

Ye and Liu	NAFLD transcriptional networks	Microarrays on 10 steatotic, 16 NASH, and 19 normal subjects	(86)

Ahrens et al.	Liver transcriptome and methylome after bariatric surgery	Microarrays on 15 NASH, 12 NAFLD, 18 obese and 18 control subjects, and 23 post-bariatric surgery	(87)

Teufel et al.	Comparison of liver transcriptomes in mouse models of NAFLD with human NAFLD or NASH	Microarrays on C57BL/6 mice, and 25 obese, 27 NAFLD, 25 NASH, and 39 normal human subjects	(88)

Lin et al.	Transcriptome in hepatocellular carcinoma (HCC)	RNA-seq on 56 paired tumor and non-tumor tissue; HBV+, HCV+, and non-viral	(104)

Burchard et al.	Liver transcriptome in HCC	Microarrays on 96 HBV-related HCC patients (paired tumor + adjacent non-tumor)	(105)

Shiraishi et al.	Transcriptome alterations and somatic mutations in liver cancer	RNA-seq on 22 paired HBV-related HCC (tumor and non-tumor tissue)	(107)

Huang et al.	Transcriptome of HBV-related HCC	RNA-seq on 10 paired HBV-related HCC (tumor and non-tumor tissue)	(108)

Tremblay et al.	RNA splicing in HCC	Reanalysis of 377 HCC samples from TCGA; HBV+, HCV+, HBV/HCV+, and non-viral	(109)

Changes in alternative RNA splicing have been detected during the development of many tissues including the brain, heart, and skeletal muscle, and more recently in liver (31). Fetal liver does not perform a metabolic function as nutrients are provided from the mother *via* the placenta. Instead the fetal liver supports hematopoiesis in the embryo (34). Hepatocytes in the embryo are proliferative but they growth arrest and differentiate after birth as the liver takes on a metabolic function (35–37). Hematopoiesis also switches from the liver to the bone marrow during late gestation. The transcription factors regulating this transition in hepatocytes and cholangiocytes have been well documented (37, 38), but many genes also show a switch in fetal-to-adult RNA isoform expression reflecting changes in alternative splicing. Bhate et al. profiled the mouse liver transcriptomes at embryonic day 18 and postnatal days 14 and 28 and at 3 months by RNA-seq (39). In addition to 4,882 changes in gene expression between e18 and adult, the authors found 529 genes that underwent a change in RNA splicing and 214 genes that underwent a change in polyadenylation. The majority of these changes in alternative splicing were conserved between mouse liver and in human fetal (22 weeks) and adult (51 years) liver tissue.

A more extensive study by Peng et al. profiled mouse liver transcriptomes at embryonic day 18, and postnatal days 0, 1, 3, 5, 10, 15, 20, 25, 30, 45, and 60 of mouse liver development (40, 41). They found 7,289 genes that were differentially expressed at some point during development, and 829 of these had multiple annotated splicing variants with 90 being differentially expressed. In addition, they found evidence for 2,383 novel splice isoforms, of which 1,455 were detected at multiple times suggesting that there is a great deal of liver transcript information yet to be annotated. As might be expected, both studies indicated that genes associated with amino acid, fatty acid, cholesterol, bile, glucose, steroid, urea, and drug metabolism were upregulated in adult liver, whereas those associated with hematopoiesis, DNA repair and metabolism, cell cycle, and chromosome reorganization were downregulated. The changes in alternative splicing were not the result of altered cell populations in the liver as the majority (88%) were still observed in purified hepatocytes (39). A number of splicing factors decreased in expression, including *Celf1, Celf2, Mbnl1, Ptbp1, Srsf1, 2, 3, 4, 6, 7, and 10, Hnrnpa1*, and *Hnrnph*, but *Esrp2* was increased in both studies.

A number of these genes have been studied *in vitro*, knocked out in the whole animal or deleted in hepatocytes (Table 2). Surprisingly, the *Mbnl* and *Celf* family proteins were identified in fetal liver. These proteins are expressed highly in muscle and have been studied extensively for their involvement in myotonic dystrophy (MD) (42–44). The whole-body knockout of *Mbnl1* causes muscle and eye abnormalities reminiscent of MD (45). Subsequently, two studies reported that loss of *Mbnl2* had no muscle phenotype but a third reported myotonia (46–48). Interestingly, one *Mbnl2* knockout mouse had a brain phenotype with impaired hippocampal plasticity and synaptic transmission consistent with high-level expression of *Mbnl2* in the brain (48). Loss of neither family member is associated with liver defects, however, but MBNL1 has been reported to regulate hematopoiesis in the fetal liver (49) potentially by regulating splicing of the *Ndel1* gene. In contrast, CUGBP1 (*Celf1*) is highly expressed in the liver but its effects appear unrelated to its role as a splicing factor, but rather are due to its role as a translational regulator as it forms a complex with eIF2 to support translation of proteins involved in liver function and regulates hepatic stellate cell activation (50–55).

**Table 2:** Genetic manipulation of RNA-binding proteins *in vivo*.

Gene	Class	Model	Phenotype	Reference
Celf1	CELF/BRUNOL family	Homozygous knockout	No liver phenotype/growth retardation? No assessment of splicing	(50)
		Transgenic overexpression	Hepatocyte proliferation in young livers. Myotonia and dystrophic muscle histology. Altered splicing	(51, 55)

Esrp2	RBM family	Homozygous knockout	Increased proliferation, diploid and tetraploid hepatocytes, smaller hepatocytes, no metabolic changes, or liver damage. Altered splicing	(39)

Hnrnpa1	HNRNP family	Homozygous knockout	Perinatal lethality. Muscle developmental defects. Impaired cardiac function. Altered splicing	(58)

Mbnl1	Zn-finger protein	Homozygous knockout	No liver phenotype, muscle and eye abnormalities characteristic of myotonic dystrophy. Altered splicing	(45)

Mbnl2	Zn-finger protein	Homozygous knockout	No liver phenotype, defects in spatial memory, abnormal REM sleep. Altered splicing	(46–48)

Ptbp1	HNRNP family	Homozygous knockout	Embryonic lethal. No assessment of splicing	(60, 61)

Slu7	Zn-finger protein	AAV-shRNA knockdown in liver	Reduced gluconeogenesis, insulin resistance, enhanced glucose uptake and glycolysis, hepatocyte proliferation, dyslipidemia. Altered splicing	(111)

Srsf1	SR protein family	Homozygous knockout	Embryonic lethal postimplantation. No assessment of splicing	(66)
		Hepatocyte knockout	No liver phenotype. No assessment of splicing	(57)
		Cardiomyocyte knockout	Excitation coupling defects. Hypertrophic cardiomyopathy. Death due to heart failure	(66)

Srsf2	SR protein family	Homozygous knockout	Embryonic lethal postimplantation. No assessment of splicing	(56)
		Hepatocyte knockout	Apoptosis, liver damage, liver failure. Altered splicing	(57)
		Cardiomyocyte knockout	Dilated cardiomyopathy. Stress-induced death. No assessment of splicing	(56)

Srsf3	SR protein family	Homozygous knockout	Embryonic lethal at blastocyst stage. No assessment of splicing	(67)
		Hepatocyte knockout	Metabolic dysfunction, steatosis, fibrosis, apoptosis and proliferation, liver damage, altered ploidy, hepatocellular carcinoma. Altered splicing	(68, 110)

Srsf10	SR protein family	Homozygous knockout	Late embryonic lethal with cardiac hypertrophy and liver degeneration. Altered splicing	(80)
		Heterozygous knockout	Increased VLDL secretion and plasma triglycerides. Altered splicing	(79)

Mice with complete deletion of Srsf2 die just after embryo implantation but mice with hepatocyte-specific deletion of *Srsf2* are viable and have normal size at birth (56, 57). The mice fail to thrive, however, and die by 2–3 weeks of age. The cause of death is liver failure. In contrast, hepatocyte-specific deletion of *Srsf1* did not have a phenotype and the mice were healthy. Livers in the newborn *Srsf2* KO mice appear normal in size and color but by day 11 the livers are pale and firmer. Histologically, the livers show hepatocyte ballooning with periportal fibrosis and inflammation. The liver failure is likely due to the lack of proliferation of hepatocytes in the neonatal liver, and increased apoptosis possibly due to endoplasmic reticulum and oxidative stress. Metabolically, the knockout livers show steatosis and lack glycogen. RNA-seq analysis indicated that the mice livers have altered cholesterol and bile homeostasis as SRSF2 stimulates expression of liver transcription factors *Srebp1c, Cebpa, Ppara, Nr1i3* (CAR1), *Nr1h4* (FXR), *Mlxipl* (CHREBBP), and *Foxa2*. Thus, SRSF2 has effects on liver RNA splicing that are not compensated by other SR proteins, unlike the role of SRSF1 that appears redundant.

Homozygous deletion of *Hnrnpa1* causes perinatal lethality within 30 min of birth because of muscle developmental defects. Death was due to cardiac dysfunction with higher blood pressure and heart rate, but defects were also observed in smooth and skeletal muscle (58). No liver phenotype was reported. The polypyrimidine tract binding protein PTBP1 (HNRNPI) binds to intronic sequences upstream of the 3′ splice site and represses splicing of pre-mRNAs (59). The effect of PTBP1 on liver function *in vivo* has not been studied as the homozygous deletion of *Ptbp1* is embryonic lethal at the implantation stage (60, 61). In HepG2 hepatoma cells, however, PTBP1 modulates splicing of multiple genes involved in cholesterol synthesis and uptake including *LDLR, MVK, HMGCS1*, and *PSCK9*. It also regulates splicing of the fatty acid desaturase genes 2 and 3 (*FADS2* and *3*) that are involved in fatty acid elongation and unsaturation (62, 63). Consequently, omega-3 and omega-6 poly-unsaturated fatty acids were reduced following *Ptbp1* knockdown, but saturated and mono-unsaturated fatty acids were not altered. Interestingly, PTBP1 is upregulated during hepatitis B virus infection and reduces expression of the proapoptotic form of FAS, which may contribute to the survival of infected hepatocytes (64).

Changes in alternative splicing during the mesenchymal to epithelial differentiation have been attributed to the splicing factors ESRP1 and ESRP2 (65). Expression of *Esrp2* was increased in the adult liver and ablation of *Esrp2* led to impaired adult splicing patterns implicating this splicing factor in the fetal-to-adult transition in hepatocytes (39). The livers did not show changes in morphology, however, nor did they display signs of liver damage, or any alterations in lipid, cholesterol, or glucose metabolism.

Other splicing factors have also been implicated in hepatocyte differentiation. Mice with complete loss of *Srsf1* or *Srsf3* die during early embryogenesis, but mice carrying a hepatocyte-specific deletion of *Srsf1* or *Srsf3* are viable (66, 67). The hepatocyte-specific deletion of *Srsf1* did not show an overt liver phenotype but loss of *Srsf3* caused impaired hepatocyte maturation (57, 68). The impaired differentiation was consistent with mis-splicing of *Hnf1a* that is critical for liver development, leading to reductions in other liver-enriched transcription factors including HNF6α (*Onecut1*), HNF3α (*Foxa3*), and C/EBPα. Consequently, the livers continued to express fetal markers such as α-fetoprotein (*Afp*) and *H19*. The impaired differentiation was associated with disrupted hepatic architecture characterized by large irregular hepatocytes, with compressed sinusoidal spaces and bile canaliculi, and reduced binuclearity. Interestingly, expression of *Esrp2* is significantly reduced in the *Srsf3* knockout, which may partly explain the impaired differentiation phenotype. The entire phenotype cannot be explained by loss of *Esrp2*, however, as *Esrp2* ablated mice do not show changes in liver morphology or histology. Loss of *Srsf3* also causes alterations in glucose and lipid homeostasis characterized by reduced glycogen storage, fasting hypoglycemia, increased insulin sensitivity, and reduced cholesterol synthesis although the target genes are distinct from those altered in the *Srsf2* knockout. Like the *Srsf2* knockout, loss of *Srsf3* causes endoplasmic reticulum stress, hepatocyte apoptosis and proliferation, and liver damage but did not cause the liver failure seen in the *Srsf2* knockout.

## Alternative Splicing and Fatty Liver

Overnutrition and obesity leads to non-alcoholic fatty liver disease (NAFLD) and its more severe form non-alcoholic steatohepatitis (NASH) (69, 70). These metabolic disturbances are becoming more common in the general population due to the current obesity epidemic (71–73). Both NAFLD and NASH are associated with the metabolic syndrome and insulin resistance, and are risk factors for type 2 diabetes, non-alcoholic liver cirrhosis, and for the development of hepatocellular carcinoma (HCC) (74, 75). So understanding the changes that occur in the fatty or NASH liver is important to elucidate mechanisms underlying the heightened risk for subsequent disease progression. Transcriptome profiling by microarray has been performed in humans with NAFLD (76, 77). While this allows gene expression changes to be monitored, most studies do not address changes in RNA alternative splicing (78). Toward the goal of understanding changes in splicing, Pihlajamaki et al. profiled gene expression in liver samples from insulin-resistant humans with obesity (79). The top-ranked pathway downregulated in obese liver samples related to RNA processing and splicing. A number of splicing factors were decreased including *SRSF10, SRSF7, SF3A1, SRSF2, SFPQ*, and *HNRNPs A1, K, D*, and *H*. The authors showed that knockdown of *SRSF10* increased lipogenesis *in vitro* in HepG2 cells and that heterozygous loss of *Srsf10* in mice increased plasma triglycerides due to increased secretion of VLDL and mis-splicing of the lipid storage protein LIPIN-1 (*Lpin1*). Homozygous deletion of *Srsf10*, however, causes embryonic lethality with liver degeneration (80). This was the first example of how a change in RNA splicing could cause a change in lipid metabolism in the obese liver. SRSF10 may also regulate the splicing of the scavenger receptor class B, member 1 gene (SCARB1) that encodes the SR-BI and SR-BII proteins that mediate reverse cholesterol transport (81). The loss of SRSF10 in obesity remains controversial, however, as it was not seen in another study (82).

Another large microarray study examined liver gene expression in 72 subjects with mild or advanced NAFLD, 10 normal liver, and 17 subjects with HBV-associated liver failure (77, 83, 84). Ninety-two splicing factor genes were altered in this dataset with 30 splicing factors being altered in either mild or advanced NAFLD. Many of these were also identified in the Pihlajamaki study. Another study has shown a decrease in SRSF4 in NASH (85). A systems biology weighted gene co-expression network analysis of 16 human NASH, 10 NAFLD, and 19 normal liver samples identified a highly significant module (*p* < 2 × 10^−6^) associated with RNA processing (86). These changes are not in all datasets, however, as a German study in 45 morbidly obese subjects with NAFLD or NASH did not show alterations in splicing factor expression (87, 88). Studies in mice have shown similar changes in the expression of splicing factors in diet-induced obesity and NASH models (79, 85, 88, 89). So NAFLD and NASH are associated with changes in RNA splicing factor expression in the liver, and this likely contributes to alterations in RNA splicing. Transcriptome profiling by RNA-seq could potentially provide a measure of RNA splicing although such an approach has not been published. It will be interesting to see whether these alterations in RNA splicing can contribute to the pathophysiology.

## Alternative Splicing and HCC

Worldwide, more than 700,000 people are diagnosed and 600,000 people die each year of liver cancer. HCC is the most common primary liver cancer (70–85%) (90) and usually arises after years of liver disease and inflammation (91) either due to chronic hepatitis B or C virus (HBV/HCV) infection (92), or alcoholic and non-alcoholic cirrhosis. The relative importance of these HCC subtypes depends on geography. HCC in HBV/HCV endemic regions in Asia and Africa is 80–90% virus associated, compared to only 20–50% of HCC in the US (93–95). Approximately 15–25% of HBV-infected individuals will develop chronic liver disease including cirrhosis, liver cancer, or failure, and 5–20% of HCV-infected individuals develop cirrhosis. A large majority (80%) of patients with HCC have cirrhosis, so cirrhosis is a major risk factor, but only 8% of patients with cirrhosis will develop HCC (96). In addition to chronic alcoholism, cirrhosis can have viral or metabolic causes (97–99), and alcohol use by at-risk individuals substantially increases the risk of cirrhosis and HCC. From a metabolic standpoint, obesity, NAFLD, and NASH are all risk factors for cirrhosis (75).

Alterations in RNA splicing in cancer have been known for over 30 years (11, 100, 101). Profiling the molecular alterations that occur in HCC has uncovered a number of targets with altered RNA splicing including the *DNMT3b, AURKB, MDM2, TENSIN2, MAD1, KLF6, SVH, TP73, TP53*, and *FN1* genes (102). Many of these changes have been shown to have functional effects to promote proliferation, prevent apoptosis, and support transformation in cell culture experiments. RNA splicing is also important for HBV and HCV viral expression, and many viruses hijack the cellular splicing machinery to allow splicing of viral RNAs (103). More recent studies have utilized high-throughput technologies to survey the HCC transcriptome (104–107). A 2011 study sequenced the transcriptomes of 10 matched pairs of cancer and non-cancerous liver tissue from HBV-infected individuals (108). A total of 1,378 differentially expressed genes were identified in HCC, but more surprisingly 24,338 exons were differentially expressed, and the vast majority of differentially expressed genes also contained differentially expressed exons. A recent study utilized the RNA-seq data available through the TCGA database. Sequence data from 377 liver samples were reanalyzed to assess alterations in RNA splicing, uncovering ~45,000 alternative splicing events (109). These events were further filtered allowing the identification of 3,250 transcripts from 2,051 genes whose expression was altered in HBV-associated HCC, 1,380 transcripts from 907 genes that were altered in HCV-associated HCC, and 1,517 genes altered in non-viral HCC. Of these transcripts, 1,336 were shared by at least two groups. The authors also assessed splicing factor expression in these samples and found altered expression of 26 splicing factors, including ESRP2, SRSF2, CELF2, MBNL1, HNRNPA1, and HNRNPH, that were found altered in hepatocyte maturation study by Bhate et al. (39), that is consistent with oncofetal transformation. These studies are likely underestimates of the true dysregulated RNA splicing as most approaches rely on databases of known annotated RNA isoforms, so will exclude reads that do not correspond to known splicing events.

## Conclusion and Future Perspectives

Although gross alterations in gene expression have been documented in nearly every disease state, recent data indicate that more subtle qualitative changes also occur, which may be just as important in disease pathogenesis. Recent high-throughput technologies are allowing a reassessment of these transcriptional changes with much higher resolution, providing a comprehensive documentation of individual transcript isoform identity and relative expression. These isoforms ultimately encode different proteins that could influence cellular function. Do these changes play a causal role in disease pathogenesis or are they simply a side effect of the disease? Traditionally, cancer was considered a disease of the genome and many of the changes in RNA splicing were thought to be a result of global alterations in gene expression in the cancer genome. Recent data, however, are indicating that subtle alterations in RNA splicing are observed in early disease, long before genomic alterations have occurred, and these alterations may play a role in predisposition to later disease. Data from mouse studies have suggested that altered splicing may cause cancer. Overexpression of the SR proteins SRSF1, SRSF3, and TRA2β (SRSF10) transforms fibroblasts and accelerates tumor growth in nude mice, and the proteins have been found to be elevated in certain cancers suggesting that they are proto-oncogenes. Interestingly, SRSF3 loss in hepatocytes also leads to liver cancer in mice (110), and SRSF3 is reduced in human HCC (111), so the properties of individual splicing factors may depend on cellular context. Aside from the SR proteins, other RNA-binding proteins that have been implicated as hnRNP proteins hnRNPA1, hnRNPA2, hnRNPH, and hnRNPI (PTB) are overexpressed in certain cancers (24, 100, 112–114), and knockdown of the proteins causes apoptosis *in vitro*. Overexpression of the zinc-finger protein MBNL2 in HCC correlates with smaller lower grade tumors and inhibits tumor growth and invasion in mice (115). Somatic mutations in splicing factor genes have also been found in cancers, the most frequently mutated being *SF3B1, U2AF1, SRSF2*, and *ZRSR2* (116). Thus, dysregulation of RNA splicing may precede and predispose to carcinogenesis, and changes in splicing may be an early event in cancer initiation and warrant further investigation. Further studies testing individual transcript changes will be required to complete our understanding of the subtleties of gene expression that underlie early disease pathogenesis.

## Author Contributions

NW conceived of and wrote the review.

## Conflict of Interest Statement

The author declares that the research was conducted in the absence of any commercial or financial relationships that could be construed as a potential conflict of interest.
